# Restoring Spinal Noradrenergic Inhibitory Tone Attenuates Pain Hypersensitivity in a Rat Model of Parkinson's Disease

**DOI:** 10.1155/2016/6383240

**Published:** 2016-09-26

**Authors:** Lei-Fang Cao, Xiao-Yan Peng, Ya Huang, Bing Wang, Feng-Ming Zhou, Ruo-Xiao Cheng, Li-Hua Chen, Wei-Feng Luo, Tong Liu

**Affiliations:** ^1^Department of Neurology, The Second Affiliated Hospital of Soochow University, Suzhou, Jiangsu 215004, China; ^2^Suqian First Hospital, Suqian, Jiangsu 223800, China; ^3^Institute of Neuroscience, Soochow University, Suzhou, Jiangsu 215123, China; ^4^Jiangsu Key Laboratory of Preventive and Translational Medicine for Geriatric Diseases, School of Public Health, Soochow University, Suzhou, Jiangsu 215123, China

## Abstract

In the present study, we investigated whether restoring descending noradrenergic inhibitory tone can attenuate pain in a PD rat model, which was established by stereotaxic infusion of 6-hydroxydopamine (6-OHDA) into the bilateral striatum (CPu). PD rats developed thermal and mechanical hypersensitivity at the 4th week after surgery. HPLC analysis showed that NE content, but not dopamine or 5-HT, significantly decreased in lumbar spinal cord in PD rats. Additional noradrenergic depletion by injection of N-(2-chloroethyl)-N-ethyl-2-bromobenzylamine (DSP-4) aggravated pain hypersensitivity in PD rats. At the 5th week after injection of 6-OHDA, systemic treatment with pharmacological norepinephrine (NE) precursor droxidopa (L-DOPS) or *α*2 adrenoceptor agonist clonidine significantly attenuated thermal and mechanical pain hypersensitivity in PD rats. Furthermore, application of norepinephrine (NE) and 5-hydroxytryptamine (5-HT) reuptake inhibitors duloxetine, but not 5-HT selective reuptake inhibitors sertraline, significantly inhibited thermal and mechanical pain hypersensitivity in PD rats. Systemic administration of Madopar (L-DOPA) or the D2/D3 agonist pramipexole slightly inhibited the thermal, but not mechanical, hypersensitivity in PD rats. Thus, our study revealed that impairment of descending noradrenergic system may play a key role in PD-associated pain and restoring spinal noradrenergic inhibitory tone may serve as a novel strategy to manage PD-associated pain.

## 1. Introduction 

Parkinson's disease (PD) is a progressive and incurable neurodegenerative disorder affecting 1% of people over 65 years and recognized clinically by its motor symptoms, such as bradykinesia, rest tremor, rigidity, and postural instability [1–3]. In recent years, nonmotor symptoms in PD patients, including neuropsychiatric symptoms, pain, sexual difficulties, sleep disorders, and gastrointestinal symptoms, have been received much more attention, because they significantly reduced quality of life in PD patients if left untreated [4–8]. Pain is a common nonmotor symptom and has a disabling effect on quality of life in PD patients, affecting about 60% to 85% of PD patients [9–12]. Chronic pain remains underrecognized and poorly treated in PD patients. Of those, musculoskeletal pain as the most common form was in about 70%, dystonic pain in 40%, radicular-neuropathic pain in 20%, and central pain in 10% [13]. Pain in PD patients may even occur in advance of the onset of motor symptoms [12], and nonmotor symptoms were more prominent in PD patients with pain compared to that of PD patients without pain [14]. Treatment of motor symptoms with dopaminergic medication or application of deep brain stimulation (DBS) in subthalamic nucleus may produce analgesic effects [15–18], but these treatments become ineffective as PD progresses [19]. So far, there are no guidelines or standard remedies for the management of PD-associated pain [20]. The management of PD-associated pain remains challenging and a major hurdle to treat PD-associated pain is the lack of information about the mechanisms underlying PD pain. Thus, it is necessary and urgent to reveal the pathogenesis of PD-associated pain by using animal models of PD.

It is well known that degeneration of dopaminergic neurons in the substantia nigra (SN), where the major dopaminergic neurons projected to the motor-regulating nucleus in the basal ganglia, contributes to the motor symptoms [1, 21] and pain in PD patients [6, 22, 23]. However, dysfunction of other neurotransmitter systems, including serotonergic, noradrenergic, and cholinergic systems, contributes to the pathogenesis of nonmotor symptoms in PD patients [24–27]. The presence of Lewy pathology and loss of cells in the locus coeruleus (LC) was noted to appear almost always in the PD progress [28]. Because LC contains the major group of noradrenergic neurons in the brain, loss of norepinephrine (NE) was even earlier and greater extent than loss of DA in PD patients [29]. Deficiency of NE accounts for many features of autonomic impairments, such as orthostatic and postprandial hypotension, and some other nonmotor symptoms, such as cognitive impairment, sleep impairment, and depression [26, 30, 31]. However, whether NE deficiency contributes to the pathogenesis of PD-associated pain is still unclear.

The descending noradrenergic projection strongly innervates the spinal cord dorsal horn, where NE could modulate nociceptive information processing [32, 33]. There was extensive evidence indicated that administration of NE in the spinal cord produced antinociceptive effects due to action on *α*2-adrenoceptors (*α*2AR) [34–36], suggesting descending noradrenergic inhibitory tone regulates pain processing. The activation of descending noradrenergic system by pain stimulation forms a negative feedback system that suppresses pain signals in spinal cord [32]. Under neuropathic pain condition, dysfunction within descending noradrenergic system as one of key disinhibition mechanisms has been implicated in the pathogenesis of chronic pain [32]. Strategies to restore the descending noradrenergic inhibitory tone, for example, the use of NE reuptake inhibitors, have been demonstrated to be effective in alleviating neuropathic pain [36, 37]. Based on the aforementioned NE deficiency in PD patients, we hypothesized that PD-associated pain may be attributed to loss of descending noradrenergic inhibitory projections.

The aims of the present study were to examine the role of descending noradrenergic system in PD-induced pain hypersensitivity and to test whether restoring the descending noradrenergic inhibitory tone could alleviate PD-induced pain. A PD rat model was established by 6-hydroxydopamine (6-OHDA) injection in bilateral striatum (CPu) and validated by immunostaining and behavioral testing. We found that the level of NE, but not dopamine and 5-HT, was significantly decreased in lumbar spinal cord after surgery by high-performance liquid chromatography (HPLC) analysis, suggesting impaired descending noradrenergic inhibitory tone may play important roles in PD-associated pain. Furthermore, systemic application of droxidopa and duloxetine to restore spinal noradrenergic inhibitory tone had analgesic effects on PD-associated pain. Thus, our results may have important clinical implication for management of PD-associated pain.

## 2. Materials and Methods

### 2.1. Animals

Adult male Sprague-Dawley (SD) rats (weighing 200–250 g) were obtained from the Shanghai SLAC Laboratory Animal Co. Ltd. All animal experiments were approved by the Ethics Committee for Animal Experiments of the Soochow University (Suzhou, China). All procedures were in agreement with the guidelines of International Association for the Study of Pain (IASP) [38] and efforts were made to minimize the number of animals used and suffering. Animals were housed in groups of three to four rats per cage with food and water available* ad libitum* and kept in controlled room temperature (22 ± 2°C) and humidity (60–80%) under a 12 h light/dark cycle.

### 2.2. Drugs and Administration

The following drugs were used: 6-hydroxydopamine (6-OHDA; H4381; Sigma-Aldrich, MO, USA) was dissolved in 0.9% NaCl, supplemented with 0.03% ascorbic acid. N-(2-Chloroethyl)-N-ethyl-2-bromobenzylamine (DSP-4; C8417; Sigma-Aldrich, MO, USA) and 5,7-dihydroxytryptamine (5,7-DHT; 37970; Sigma-Aldrich, MO, USA) were dissolved in 0.9% NaCl containing 0.1% of ascorbic acid. Formalin (F8775, Sigma-Aldrich, USA) was dissolved in 0.9% NaCl. Duloxetine (IN46285, Eli Lily and Company, Indianapolis, USA) was dissolved with 10% dimethylsulfoxide. Madopar (L-DOPA; Shanghai Roche Pharmaceutical Co. Ltd, Shanghai, China), pramipexole (Boehringer Ingelheim, Germany), droxidopa (Chongqing Shenghuaxi Pharmaceutical Co. Ltd, Chongqing, China), sertraline (Pfizer Inc., Liaoning, China), and clonidine (C7897; Sigma-Aldrich, MO, USA) were dissolved in saline.

The dosages used in the present study are as follows: Madopar (L-DOPA; i.p. 15 mg/kg); pramipexole (a D2/D3 receptor agonist; i.p. 1 mg/kg); droxidopa (a prodrug to NE; i.p. 10 and 20 mg/kg); clonidine (*α*_2_ receptor agonist; i.p. 200 *μ*g/kg); duloxetine (a 5-HT and NE reuptake inhibitor; i.p. 10 mg/kg); sertraline (a selective serotonin reuptake inhibitor; i.p. 10 mg/kg). All the drugs were administered at a volume of 0.6–1 mL. The control group received the same volume of saline or 10% dimethylsulfoxide.

### 2.3. Establishment of 6-OHDA-Induced PD Model

To develop a rat PD model, the rat received a stereotaxic injection of 6-OHDA into the bilateral striatum as reported previously [39]. Briefly, rats were anesthetized by 4% chloral hydrate (i.p. 10 mL/kg) and microinjection was performed via a 10 *μ*L Hamilton syringe using stereotaxic apparatus (David Kopf Instruments, Tujunga, CA, USA) with the following coordinates relative to bregma, according to the stereotaxic atlas of Paxinos and Watson: anteroposterior (AP), –1.0 mm; mediolateral (ML), ±3.5 mm; dorsoventral (DV), –4.5 mm. 6-OHDA (10 *μ*g/5 *μ*L, each side) were infused in the flow rate of 0.5 *μ*L/min and the needle was left in the place for additional 5 min at the end and then withdrawn at a rate of 1 mm/min. Posterior placement of the injectors is determinant factor for sparing a part of DA ascending fibers and therefore to circumvent severe alterations of motor function.

### 2.4. The Noradrenergic System Lesions by DSP-4

To selectively induce the lesion of the noradrenergic system, we used N-(2-chloroethyl)-N-ethyl-2-bromobenzylamine (DSP-4), which is a competitive inhibitor of norepinephrine uptake and selectively degenerates noradrenergic neurons [40, 41]. The rats received a bilateral intracerebroventricular (ICV) injection of DSP-4 (100 *μ*g/4 *μ*L in each side) by using microinjection pump. Bilateral infusions were done to maximize delivery of the solution into the ventricles and to ensure symmetrical distribution of the neurotoxin to all portions of the noradrenergic system. The sites were following coordinates relative to bregma: AP: −0.6 mm, ML: ±1.5 mm, and DV: −5.0 mm. At the end of microinjection, the needle was left in the place for additional 5 min and withdrawn at a rate of 1 mm/min.

### 2.5. The 5-HT System Lesions by 5,7-DHT

To explore the functions of 5-hydroxytryptamine (5-HT) system in PD-associated pain, we exerted 5,7-dihydroxytryptamine (5,7-DHT, 100 *μ*g/4 *μ*L in each side) to destroy 5-HT system as previously reported [42, 43]. Surgical procedures were the same as described in Section 2.4; only the rats received 5,7-DHT rather than DSP-4. The sham animals received vehicle via the same procedure.

### 2.6. Behavioral Tests

All experiments were conducted during the light phase of the cycle (between 8:00 am and 6:00 pm). All animals were handled for 3–5 days (daily 30 min) before behavioral tests. All the behavioral tests were done in a blind manner to the drug treatment.

#### 2.6.1. Hargreaves Test

For testing heat sensitivity, we put the rats in plastic boxes and measured the hindpaw withdrawal latency to radiant heat apparatus (IITC Life Science) [44]. Rats were placed on a glass floor maintained at 25°C in a clear plastic chamber. We focused on a radiant heat source, which was located under the glass floor, onto the plantar surface of the hindpaw. We measured paw withdrawal latency 2-3 times for each hindpaw, and we used the mean of the results for analysis. Five min of rest was allowed between trials. We set a cut-off of 20 s to prevent potential tissue.

#### 2.6.2. Von Frey Filament Test

To test mechanical hypersensitivity, we determined the mechanical sensitivity of hindpaws by using series of von Frey filaments (4 g, 8 g, and 15 g) as previously reported [45]. The rats were placed on a metal mesh floor and von Frey filaments were applied from underneath the floor. The plantar surface of hindpaw was received consecutively by 10 stimulations. This was repeated twice at an interval of 10 minutes. We used response frequency to estimate the mechanical hypersensitivity.

#### 2.6.3. Formalin Test

On the 5th week after bilateral 6-OHDA lesion, low dose of formalin (20 *μ*L of 0.2%) was injected into the plantar surface of hindpaw using a 30-gauge needle as previously reported [46]. Following injection, rats was video-recorded with Sony HDR CX610E immediately by 60 minutes in a clear plastic enclosure. Number of flinches was quantified by an observer in a blinded manner.

#### 2.6.4. Rotarod Test

In order to assess the motor function, we used the rotarod apparatus [47]. Rats were pretrained, 3-4 consecutive days, with the rod rotating at an accelerating speed from 4 to 40 rpm during two minutes, until they keep a stable baseline performance. On the days of testing rats were evaluated before and on weeks 1, 2, 3, 4, and 5 after operation. Each rat had three trials, separated by 5 min and the average of the three attempts was recorded (seconds on the rod), calculated, and analyzed.

### 2.7. Western Blotting Assay

At the 5th week after 6-OHDA injection, the rats were terminally anesthetized with 4% chloral hydrate (i.p. 10 mL/kg) and transcardially perfused with saline; the corpus striatum was rapidly removed and homogenized in a lysis buffer containing a cocktail of protease inhibitors and phosphatase inhibitors for total protein extraction and assay according to the previous report. The protein concentrations were determined by BCA Protein Assay (Pierce, Rockford, IL, USA) and 21 *μ*g of proteins was loaded for each lane and subjected to SDS-PAGE. After the transfer, the blots were blocked with 5% nonfat milk in TBS and PVDF membranes were incubated overnight at 4°C with primary polyclonal antibody against the TH (rabbit, 1 : 1000; Suzhou Ruiying Biotechnology Co. Ltd, Jiangsu China). For loading control, the blots were probed with GAPDH antibody (mouse, 1 : 2000, Vazyme, Suzhou City, Jiangsu Province, China). The blots were washed and incubated in horseradish peroxidase-conjugated goat anti-rabbit or goat anti-mouse IgG secondary antibody (1 : 2000, Santa Cruz Biotechnology). Protein bands were visualized using an enhanced chemiluminescence detection kit (Pierce) and the band densities were detected and analyzed using Molecular Imager ChemiDoc XRS + System (Bio-Rad, Shanghai, China). Data from five rats were used for statistical analysis system (Bio-Rad).

### 2.8. Immunofluorescence

At the 5th week after 6-OHDA injection, the rats were terminally anesthetized with 4% chloral hydrate (i.p. 10 mL/kg) and intracardially perfused first with saline and then with 4% of the fixative solution paraformaldehyde in 0.1 M phosphate buffer (pH = 7.4). Brain was collected and postfixed in the same solution overnight. After cryoprotection through a graded series of sucrose replacements (10%, 20%, and 30% in PBS) at 4°C, each segment was embedded in OCT Compound (4853, Sakura Finetek, Torrance, USA) and stored at −80°C. Then, the substantia nigra sections were cut at the thickness of 15 *μ*m in a cryostat (CM1950, Leica Biosystems Nussloch GmbH, Germany). The tissue sections were mounted on siliconized slides for immunostaining. Nonspecific labeling was blocked by incubation in 10% normal goat serum and 0.3% Triton X-100 in PBS. After blocking, the sections were incubated over night at 4°C with the primary mouse anti-tyrosine hydroxylase (TH) monoclonal antibody (1 : 1000, Sigma, USA). After that, the sections were rinsed in PBS and incubated in Cy3-conjugated affinipure goat anti-mouse IgG (1 : 500, Jackson ImmunoResearch, West Grove, PA, USA) for 1 h at room temperature. The sections were subsequently rinsed in PBS and mounted with VECTSHIELD with DAPI (CA94010, Vector Laboratories, Inc. Burlingame, USA). Immunostained tissue sections were examined under a Zeiss fluorescence microscope AXIO SCOPE A1 (Oberkochen, Germany) and images were analyzed with NIH Image software or Adobe Photoshop.

### 2.9. HPLC Analysis for NE, 5-HT, and DA

At the 5th week after bilateral 6-OHDA-injection, animals were deeply anesthetized by isoflurane and intracardially perfused with cool saline. The lumbar spinal cord was dissected, weighed, and then homogenized with an ultrasonic homogenizer (Microsonic, Dortmund, Germany) in 400 *μ*L 0.4 mol/L perchloric acid. Samples were then centrifuged at 15000 rpm at 4°C for 10 min, filtered through the 0.22 *μ*m syringe filter, and stored at −80°C until neurochemical analysis. The concentrations of NE, 5-HT, and DA were measured by applying reverse-phase HPLC with electrochemical detection (SHIMADZU, LC-6A, Japan). The reversed phase column was YWG-C_18_, which was perfused for analysis with a mobile phase composed of 0.1 mol/L NaAc (including 0.1 mol/L EDTA-Na_2_) and 10% methanol at pH 5.1. The flow rate was 1 mL/min. The data was quantified using the area under the peaks and external standards. The obtained results were presented in ng per gram of wet tissue (ng/g).

### 2.10. Statistical Analysis

Statistical data were calculated using GraphPad Prism 6 software (La Jolla, CA, USA). All data were presented as mean ± SEM. Two-way ANOVA with Bonferroni multiple comparisons tests were used for between-groups comparisons. Student's unpaired* t*-test was used to analyze the difference between two groups.* P* value <0.05 was considered to be statistical significant.

## 3. Results

### 3.1. PD Model Was Established by Bilateral 6-OHDA Infusions into the Striatum (CPu) in Rats

**Figure 1:**
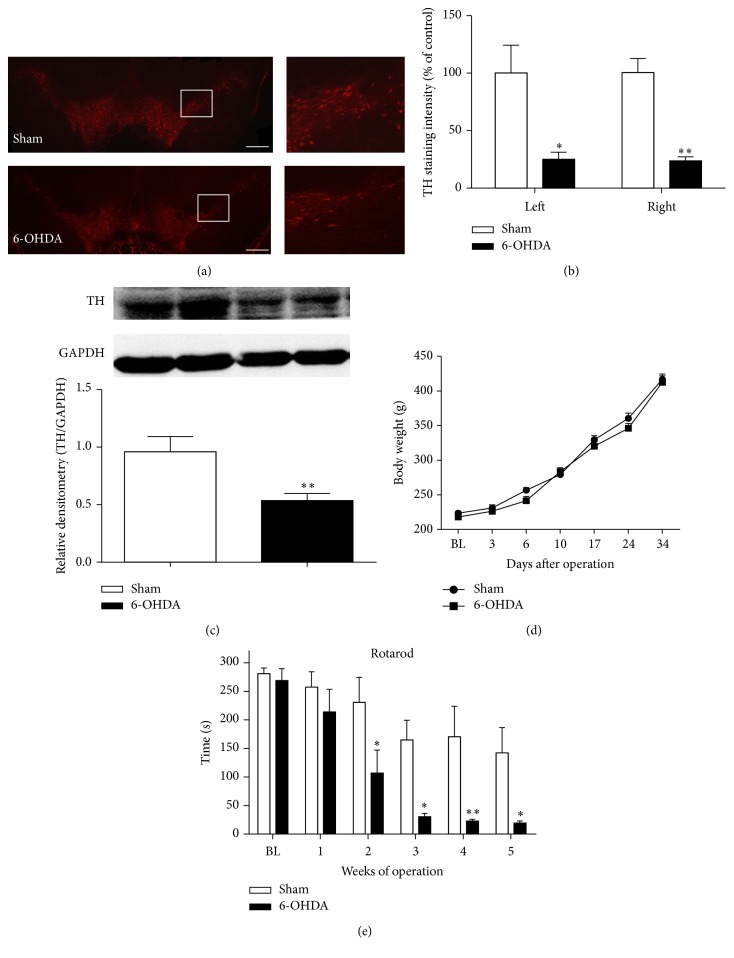
Parkinson's disease model induced by stereotaxic infusion of 6-OHDA into the bilateral striatum of rats. (a) Representative photomicrographs of coronal section showing tyrosine hydroxylase-immunoreactive neurons and fibers in the substantia nigra (SN). The right lanes of pictures are higher magnification of boxed area. Scale bar = 100 *μ*m. (b) The quantification analysis showed the TH-immunostaining intensity in SNc plus SNr (substantia nigra reticular part) at the 5th week after microinjection. Compared with the sham group, the TH-immunoreactivity was significantly decreased in the 6-OHDA-treated group (^*∗*^ *P* < 0.05, ^*∗∗*^ *P* < 0.01 compared to the sham control, *n* = 4 for each group). (c) The expression level of TH in striatum (CPu) was revealed by western blotting. TH expression in striatum (CPu) was significantly reduced in 6-OHDA-treated rats at the 5th week after operation compared to the sham control. Bottom bar graph showed the relative density of TH/GAPDH between 6-OHDA-treated group (*n* = 4) and sham group (*n* = 4). ^*∗∗*^ *P* < 0.01 compared to the sham control. (d) There was no significant difference for body weight between 6-OHDA-treated group (*n* = 10) and sham group (*n* = 10). (e) The rotarod test showed that comparing to the sham group (*n* = 5), the latency time to fall from the rod for 6-OHDA-treated rats (*n* = 8) was significantly decreased from the 2nd week after operation (^*∗*^ *P* < 0.05; ^*∗∗*^ *P* < 0.01 compared to the sham group).

As previously reported [39, 48], we used bilateral 6-OHDA infusions (10 *μ*g/5 *μ*L, each side) in the striatum (CPu) to induce striatum dopamine denervation and subsequent loss of dopaminergic neurons in the substantia nigra (SN). Parkinsonian phenotype was validated in 6-OHDA-lesioned rats using tyrosine hydroxylase (TH) quantification and behavioral testing. As shown in Figure 1, immunofluorescence analysis revealed that the relative level of TH-immunoreactive staining intensity in SN in the 6-OHDA-lesion group was decreased by about 80% comparing to the sham group (Figures 1(a) and 1(b), left: *P* = 0.01, right: *P* = 0.003, *n* = 4 rats/group). Western blotting analysis also showed that protein level of TH in 6-OHDA-lesioned group significantly decreased in the CPu by 45% compared to the sham group (Figure 1(c), *P* = 0.0012, *n* = 4 rats/group). For the body weight, there was no significant difference between 6-OHDA-lesioned and sham rats (Figure 1(d), *P* > 0.05, *n* = 10 rats/group). The rotarod test was used to examine the motor coordination in sham and 6-OHDA-lesioned rats. As shown in Figure 1(e), the time spent on the rod in the 6-OHDA-lesioned rats demonstrated a significant decrease compared to sham animals from the 2nd week after surgery (*F*_time(5,45)_ = 23.32, *P* < 0.0001; *F*_group(1,9)_ = 9.653, *P* = 0.012; *F*_time×group(5,45)_ = 2.719, *P* = 0.0314). Consistent with previous report [49], our results suggested that bilateral injection of 6-OHDA into the striatum could be used as a suitable PD model in rats.

### 3.2. Thermal and Mechanical Hypersensitivity Were Observed at the 4th Week after 6-OHDA Lesion in Rats

**Figure 2:**
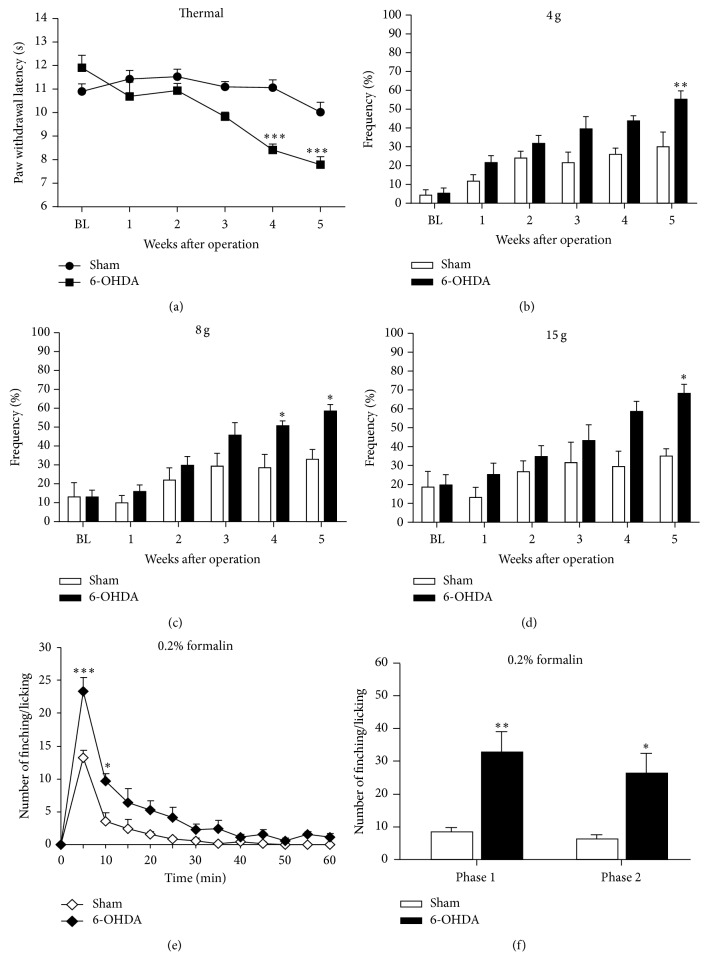
The development of pain hypersensitivity in the 6-OHDA-lesioned rats. After testing the baseline response to heat and mechanical stimuli, rats received a bilateral dorsal CPu injection of 6-OHDA or vehicle. (a) Paw thermal withdrawal threshold was significantly decreased in the 6-OHDA-treated rats (*n* = 12) than in the sham control rats (*n* = 5) on the 4th and 5th weeks after operation. ((b)–(d)) 4 g, 8 g, and 15 g von Frey filament were used to evaluate mechanical hypersensitivity. The 6-OHDA-lesioned rats (*n* = 12) showed mechanical hypersensitivity from the 4th and 5th week after the 6-OHDA lesion compared with the sham rats (*n* = 5). ^*∗*^ *P* < 0.05, ^*∗∗*^ *P* < 0.01, and ^*∗∗∗*^ *P* < 0.001 compared to the sham animals. ((e)-(f)) At the 5th week after 6-OHDA microinjection, we used the formalin test to determine the changes of chemical-induced pain. There was a significant increase in both phase 1 (0–10 min) and phase 2 (10–60 min) in 6-OHDA-treated rats (*n* = 8) compared to the sham animals (*n* = 5) (^*∗*^ *P* < 0.05, ^*∗∗*^ *P* < 0.01, and ^*∗∗∗*^ *P* < 0.001 compared to the sham animals).

To determine whether bilateral injection of 6-OHDA into the striatum was sufficient to induce changes in thermal and mechanical thresholds in rats, we used Hargreaves test and von Frey filament test to evaluate the thermal and mechanical sensitivity, respectively, in 6-OHDA-lesioned and sham rats before and after surgery. In sham group, the thermal pain threshold slightly decreased but with no significant difference compared to the baseline (Figure 2(a)). In PD patients, the heat pain threshold was decreased and spinal nociceptive threshold was altered by the measurement of nociceptive flexion reflex (NFR) [50]. Consistent with this human study, we also found the paw withdrawal latency to radiate heat was significantly decreased in the 6-OHDA-lesioned rats, at the 4th week, and lasted at least for 5 weeks after surgery (Figure 2(a)) (*F*_time(5,95)_ = 14.41, *P* < 0.0001; *F*_group(1,19)_ = 15.10, *P* = 0.0010; *F*_time×group  (5,95)_ = 6.419, *P* < 0.0001). To assess mechanical threshold in 6-OHDA-lesioned and sham rats, we used three different grams of von Frey filaments, including 4 g, 8 g, and 15 g, to determine the response frequency to mechanical stimuli. The mechanical sensitivity to von Frey stimuli was significantly increased in 6-OHDA-lesioned rats after the 4th or 5th week after surgery compared to sham rats (Figures 2(b)–2(d)) (4 g: *F*_time(5,75)_ = 20.72, *P* < 0.0001; *F*_group(1,15)_ = 6.900, *P* = 0.0191; *F*_time×group(5,75)_ = 2.146, *P* = 0.0691; 8 g: *F*_time(5,75)_ = 17.16, *P* < 0.0001; *F*_group(1,15)_ = 7.147, *P* = 0.0174; *F*_time×group(5,75)_ = 2.094, *P* = 0.0754; 15 g: *F*_time(5,75)_ = 15.19, *P* < 0.0001; *F*_group(1,15)_ = 2.604, *P* = 0.1274; *F*_time×group(5,75)_ = 3.621, *P* = 0.0055). We also used formalin test to evaluate changes of chemical-induced pain in 6-OHDA-lesioned rats at the 5th week after surgery. Compared to sham group, nocifensive responses were significantly increased in 6-OHDA-lesioned rats for both first phase (0–10 min) and second phase (10–60 min) (Figures 2(e) and 2(f)) (For 1st phase: *P* = 0.004; for 2nd phase: *P* = 0.019, *n* = 5–8).

### 3.3. NE Level Was Decreased in the Lumbar Spinal Cord in 6-OHDA-Lesioned Rats

**Figure 3:**
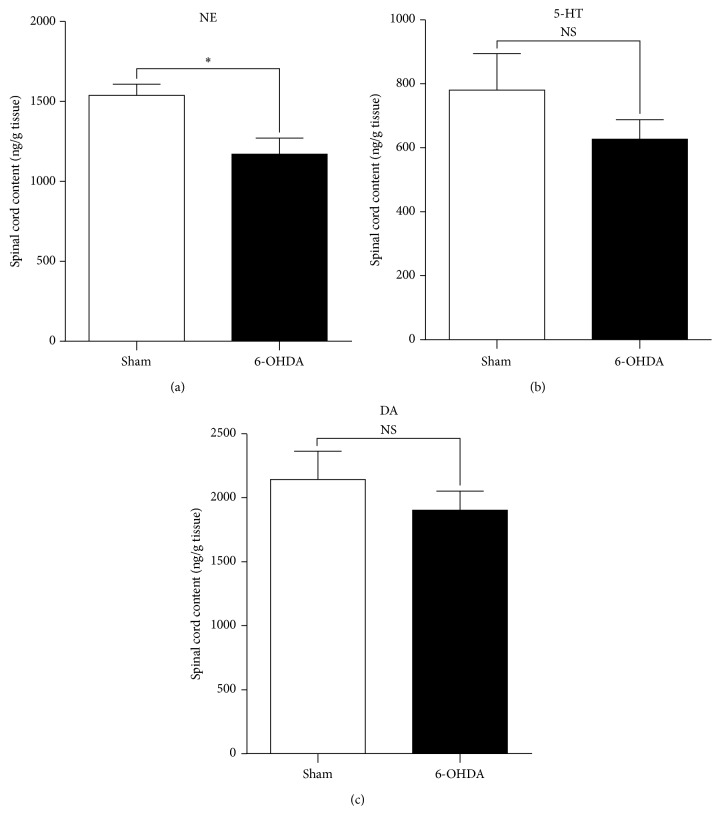
Change of NE, 5-HT, and dopamine (DA) content in the lumbar spinal cord in the 6-OHDA-lesioned rat model. Samples were collected at the 5th week after 6-OHDA-lesion and NE (a), 5-HT (b), and DA (c) content in the lumbar spinal cord were analyzed by HPLC. Compared to the sham group, the NE content significantly decreased in the 6-OHDA-lesioned animals (^*∗*^ *P* < 0.05 compared to the sham group, *n* = 4 for each group). 5-HT and DA content did not significantly change between the 6-OHDA-treated group and sham control group (*P* > 0.05 compared to the sham group, *n* = 4 for each group).

We used high-performance liquid chromatography (HPLC) analysis to determine the changes of content of dopamine (DA), NE, and 5-HT in the lumbar spinal dorsal horn in 6-OHDA-lesioned rats. As shown in Figure 3(a), NE content in the lumbar spinal cord tissues from the 6-OHDA-lesioned rats significant reduced compared to sham rats (*P* < 0.05, unpaired *t*-test). Interestingly, neither 5-HT (Figure 3(b)) nor dopamine (Figure 3(c)) changed in 6-OHDA-lesioned rats compared to those of sham rats.

### 3.4. Noradrenergic Depletion Alone Produced Pain Hypersensitivity in Normal Rats

**Figure 4:**
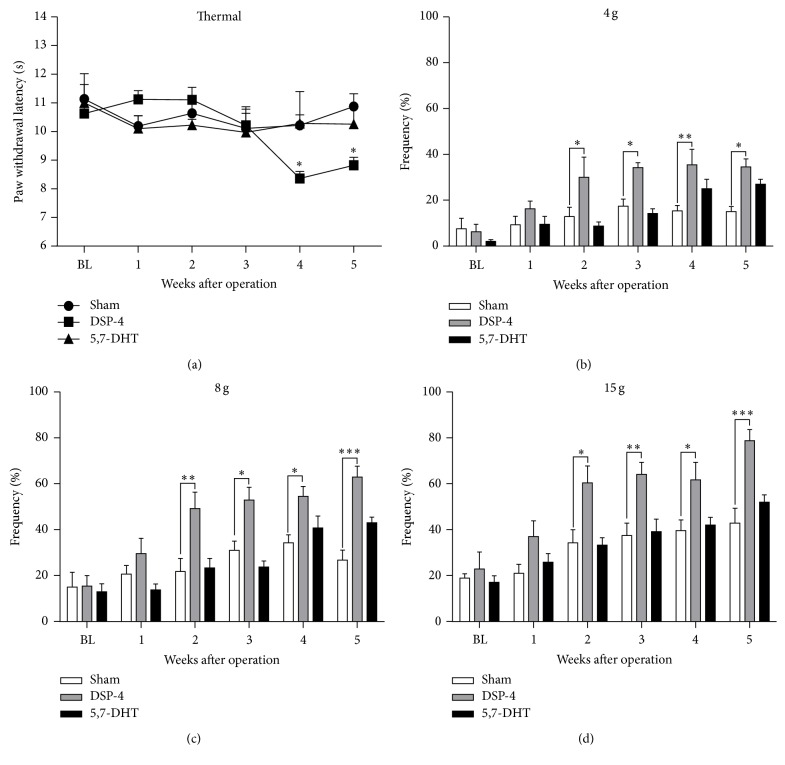
Effects of DSP-4 or 5,7-DHT injection alone on thermal and mechanical sensitivity in rats. (a) Compared to the sham group, paw thermal withdrawal threshold was decreased on the 4th week after bilateral ICV injection of DSP-4 (100 *μ*g/4 *μ*L in each side), but not 5,7-DHT (100 *μ*g/4 *μ*L in each side). ((b)–(d)) Mechanical hypersensitivity was significantly induced from the 2nd week of the end of testing after ICV injection DSP-4, but not 5,7-DHT (^*∗*^ *P* < 0.05, ^*∗∗*^ *P* < 0.01, and ^*∗∗∗*^ *P* < 0.001 compared to the sham group, *n* = 6-7 for each group).

We used stereotaxic intracerebroventricular (ICV) injection of DSP-4 (100 *μ*g/4 *μ*L in each side) to selectively reduced the noradrenaline content in brain [40, 51]. In the DSP-4-lesioned rats, the paw withdrawal latency to thermal stimulation began to decrease after the 4th week after surgery (Figure 4(a)) (*F*_time(5,55)_ = 3.867, *P* = 0.0045; *F*_group(1,11)_ = 2.356, *P* = 0.1530; *F*_time×group(5,55)_ = 3.867, *P* = 0.0045) and the mechanical hypersensitivity occurred from the 2nd week to the 5th week after surgery (Figures 4(b)–4(d)) (4 g: *F*_time(5,55)_ = 9.159, *P* < 0.0001; *F*_group(1,11)_ = 12.26, *P* = 0.005; *F*_time×group(5,55)_ = 2.71, *P* = 0.0293; 8 g: *F*_time(5,55)_ = 16.71, *P* < 0.0001; *F*_group(1,11)_ = 14.88, *P* = 0.0027; *F*_time×group(5,55)_ = 4.749, *P* = 0.0011; 15 g: *F*_time(5,55)_ = 22.36, *P* < 0.0001; *F*_group(1,11)_ = 14.95, *P* = 0.0026; *F*_time×group(5,55)_ = 2.910, *P* = 0.0211). In sharp contrast, impairment of 5-hydroxytryptamine (5-HT) system by ICV injection of 5,7-dihydroxytryptamine (5,7-DHT, 100 *μ*g/4 *μ*L in each side) did not affect the thermal and mechanical threshold in normal rats (Figure 4).

### 3.5. Additional Noradrenergic Depletion Exacerbated and Accelerated the Development of Pain Hypersensitivity in 6-OHDA-Lesioned Rats

**Figure 5:**
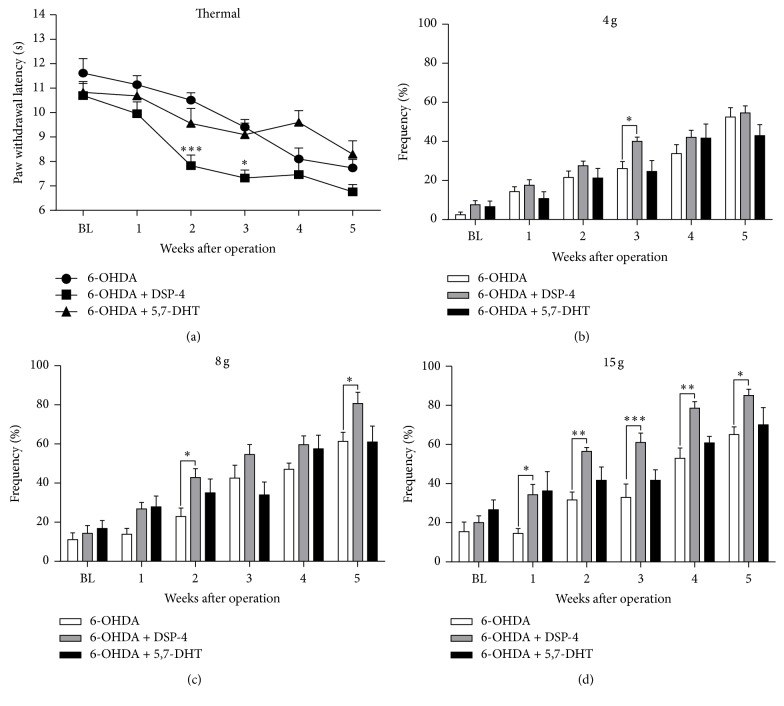
Effects of DSP-4 and 5,7-DHT on thermal and mechanical hypersensitivity in 6-OHDA-lesioned rats. (a) Compared to 6-OHDA-treated group, paw thermal withdrawal thresholds were decreased from the 2nd week after 6-OHDA plus DSP-4 injection. ((b)–(d)) Mechanical hypersensitivity was increased from the 2nd week after 6-OHDA plus DSP-4 injection and even increased from the 1st week after injection under 15 g von Frey filament (d). However, compared to 6-OHDA-treated group, coinjection of 5,7-DHT and 6-OHDA simultaneous did not further increase the mechanical hypersensitivity in rats (^*∗*^ *P* < 0.05, ^*∗∗*^ *P* < 0.01, and ^*∗∗∗*^ *P* < 0.001 compared to the sham control, *n* = 6-7 for each group).

As shown in Figure 5, we explored the effects of additional noradrenergic depletion on pain hypersensitivity in 6-OHDA-lesioned rats. Compared to the single 6-OHDA-lesioned group, the 6-OHDA plus DSP-4-treated rats showed a significant decrease of thermal paw withdrawal latency at the 2nd week after surgery (Figure 5(a)) (*F*_time(5,85)_ = 28.01, *P* < 0.0001; *F*_group(1,17)_ = 15.10, *P* = 0.0011; *F*_time×group(5,85)_ = 1.811, *P* > 0.05). Additionally, mechanical hypersensitivity also occurred earlier at the 2nd or 3rd week after surgery (Figures 5(b)–5(d)) (4 g: *F*_time  (5,85)_ = 49.80, *P* < 0.0001; *F*_group(1,17)_ = 5.742, *P* = 0.0283; *F*_time×group(5,85)_ = 0.7713, *P* > 0.05; 8 g: *F*_time(5,85)_ = 43.51, *P* < 0.0001; *F*_group(1,17)_ = 20.28, *P* = 0.0027; *F*_time×group(5,85)_ = 0.8480, *P* > 0.05; 15 g: *F*_time(5,85)_ = 52.94, *P* < 0.0001; *F*_group(1,17)_ = 24.48, *P* = 0.0001; *F*_time×group(5,85)_ = 1.892, *P* > 0.05). In contrast, additional serotonergic impairment by ICV injection of 5,7-DHT did not significantly change the development of thermal and mechanical hypersensitivity in 6-OHDA-lesioned rats (Figure 5). Thus, these results indicate that the descending NE system, but possibly not 5-HT system, plays a key role in the PD-associated pain hypersensitivity in rats.

### 3.6. Inhibitory Effects of Systemic Administration of Droxidopa and Clonidine on Pain Hypersensitivity in 6-OHDA-Lesioned Rats

**Figure 6:**
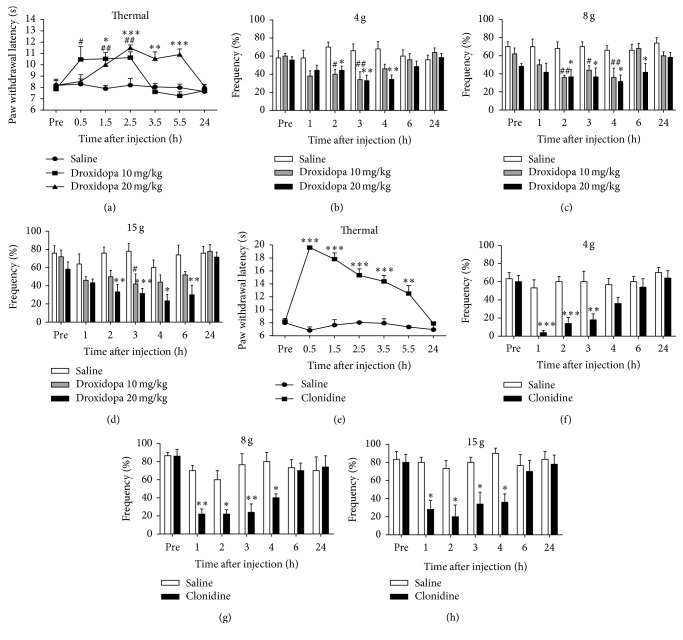
Effects of droxidopa and clonidine on thermal and mechanical hypersensitivity in 6-OHDA-lesioned rats. Rats were i.p. injected with 10 mg/kg droxidopa and 20 mg/kg droxidopa or equal volume of saline on the 5th week after surgery. ((a)–(d)) Systemic application of droxidopa significantly inhibited thermal (a) and mechanical hypersensitivity ((b)–(d)) in 6-OHDA-lesioned rats (^*∗*^ *P* < 0.05, ^*∗∗*^ *P* < 0.01, and ^*∗∗∗*^ *P* < 0.001 for 10 mg/kg droxidopa and ^#^ *P* < 0.05 and ^##^ *P* < 0.01 for 20 mg/kg droxidopa compared to the saline control, *n* = 5-6 for each group). ((e)–(h)) Systemic application of clonidine (200 *µ*g/kg, i.p.) significantly inhibited thermal (e) and mechanical hypersensitivity ((f)–(h)) in 6-OHDA-lesioned rats (^*∗*^ *P* < 0.05, ^*∗∗*^ *P* < 0.01, and ^*∗∗∗*^ *P* < 0.001 compared to the saline control, *n* = 5-6 for each group).

To further investigate the role of descending noradrenergic system in pain hypersensitivity in 6-OHDA-lesioned rats, we firstly used droxidopa (L-DOPS) to increase the level of norepinephrine in the central nervous system (CNS) [52]. Single systemic injection of L-DOPS (10 and 20 mg/kg, i.p.) significantly increased the paw withdrawal latency to thermal stimulation in 6-OHDA-lesioned rats at the 5th week after surgery (Figure 6(a)) (for 10 mg/kg: *F*_time(6,48)_ = 6.381, *P* < 0.0001; *F*_treatment(1,8)_ = 6.391, *P* = 0.0354; *F*_time×treatment(6,48)_ = 5.112, *P* = 0.0004; for 20 mg/kg: *F*_time(6,54)_ = 6.535, *P* < 0.0001; *F*_treatment(1,9)_ = 18.78, *P* = 0.0019; *F*_time×treatment(6,54)_ = 5.867, *P* < 0.0001). Application of L-DOPS significantly inhibited mechanical hypersensitivity in 6-OHDA-lesioned rats (Figures 6(b)–6(d)) (for 10 mg/kg: 4 g: *F*_time(6,48)_ = 1.454, *P* < 0.0001, *F*_treatment(1,8)_ = 6.391, *P* = 0.0354, *F*_time×treatment(6,48)_ = 5.112, *P* = 0.0004; 8 g: *F*_time(6,48)_ = 2.971, *P* = 0.0151, *F*_treatment(1,8)_ = 13.74, *P* = 0.006, *F*_time×treatment(6,48)_ = 2.382, *P* = 0.0428; 15 g: *F*_time(6,48)_ = 4.27, *P* = 0.0016, *F*_treatment(1,8)_ = 4.525, *P* = 0.0661, *F*_time×treatment(6,48)_ = 2.092, *P* = 0.0715; for 20 mg/kg: 4 g: *F*_time(6,54)_ = 0.9205, *P* = 0.4876, *F*_treatment(1,9)_ = 9.660, *P* = 0.0126, *F*_time×treatment(6,54)_ = 3.769, *P* = 0.0033; 8 g: *F*_time(6,54)_ = 2.299, *P* = 0.0476, *F*_treatment(1,9)_ = 21.76, *P* = 0.0012, *F*_time×treatment(6,54)_ = 0.9509, *P* = 0.467; 15 g: *F*_time(6,54)_ = 4.941, *P* = 0.0004, *F*_treatment(1,9)_ = 17.85, *P* = 0.0022, *F*_time×treatment(6,54)_ = 2.894, *P* = 0.0161).

We subsequently investigated the effects of a selective *α*2AR agonist clonidine on pain hypersensitivity in 6-OHDA-lesioned rats. As shown in Figure 6, systemic administration of clonidine (200 *μ*g/kg, i.p.) remarkably increased the paw withdrawal latency for radiated heat stimulation in 6-OHDA-lesioned rats at the 5th week after surgery, lasting at least 3 hours after injection of the drug (Figure 6(e)) (*F*_time(6,36)_ = 17.95, *P* < 0.0001; *F*_treatment(1,6)_ = 101.0, *P* < 0.0001; *F*_time×treatment(6,36)_ = 18.89, *P* < 0.0001). Application of clonidine also significantly suppressed mechanical hypersensitivity lasting 4 hours after drug injection (Figures 6(f)–6(h)) (4 g: *F*_time(6,36)_ = 15.16, *P* < 0.0001; *F*_treatment(1,6)_ = 8.478, *P* = 0.0269; *F*_time×treatment(6,36)_ = 7.946, *P* < 0.0001; 8 g: *F*_time(6,36)_ = 15.38, *P* < 0.0001; *F*_treatment(1,6)_ = 6.113, *P* = 0.0483; *F*_time×treatment(6,36)_ = 8.541, *P* < 0.0001; 15 g: *F*_time(6,36)_ = 7.383, *P* < 0.0001; *F*_treatment(1,6)_ = 5.816, *P* = 0.0525; *F*_time×treatment(6,36)_ = 6.125, *P* = 0.0002).

### 3.7. Inhibitory Effects of Systemic Administration of Duloxetine, but Not Sertraline, on Pain Hypersensitivity in 6-OHDA-Lesioned Rats

**Figure 7:**
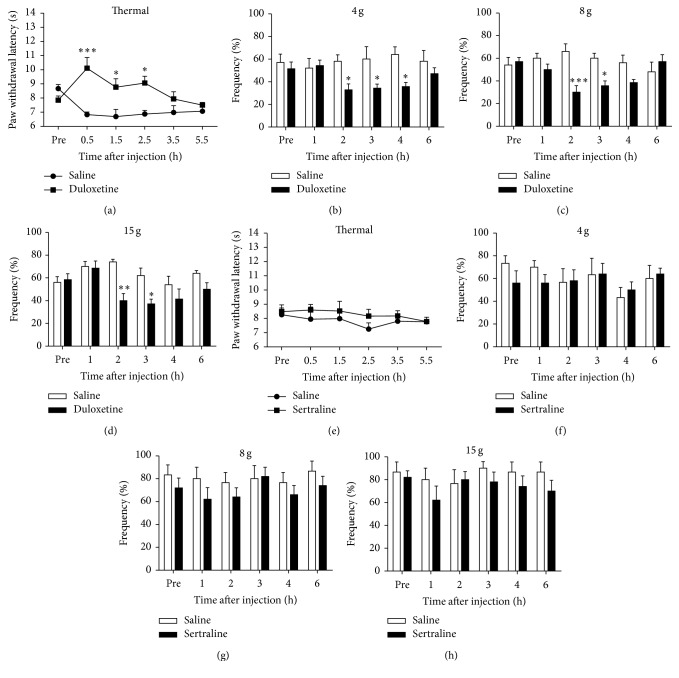
Effects of duloxetine and sertraline on thermal and mechanical hypersensitivity in 6-OHDA-lesioned rats. Rats were i.p. injected with 10 mg/kg duloxetine or equal volume of 10% dimethylsulfoxide on the 5th week after 6-OHDA microinjection. ((a)–(d)) Systemic application of duloxetine significantly inhibited thermal (a) and mechanical hypersensitivity ((b)–(d)) in the 6-OHDA-lesioned rats (^*∗*^ *P* < 0.05, ^*∗∗*^ *P* < 0.01, and ^*∗∗∗*^ *P* < 0.001 for duloxetine compared to the vehicle control, *n* = 5–7 for each group). ((e)–(h)) Systemic application of sertraline (10 mg/kg, i.p.) did not significantly affect thermal (e) and mechanical hypersensitivity ((f)–(h)) in the 6-OHDA-lesioned rats (*P* > 0.05, *n* = 5 for each group).

To investigate whether restoring descending noradrenergic inhibitory system could suppress pain hypersensitivity in 6-OHDA-lesioned rats, 6-OHDA-lesioned rats at the 5th week after surgery were treated with duloxetine, 5-HT, and NE reuptake inhibitor, to increase the levels of 5-HT and NE in spinal cord. It was found that systemic treatment of duloxetine (10 mg/kg, i.p.) significantly increased the paw withdrawal latency to thermal stimulation (Figure 7(a)) (*F*_time(5,50)_ = 1.764, *P* = 0.1374; *F*_treatment(1,10)_ = 30.18, *P* = 0.0003; *F*_time×treatment(5,50)_ = 4.506, *P* = 0.018). Application of duloxetine significantly inhibited mechanical hypersensitivity compared with saline in 6-OHDA-lesioned rats (Figures 7(b)–7(d)) (4 g: *F*_time(5,50)_ = 124, *P* = 0.3598; *F*_treatment(1,10)_ = 5.39, *P* = 0.0427; *F*_time×treatment(5,50)_ = 3.59, *P* = 0.075; 8 g: *F*_time(5,50)_ = 1.089, *P* = 0.3780; *F*_treatment(1,10)_ = 9.645, *P* = 0.0111; *F*_time×treatment(5,50)_ = 5.424, *P* = 0.005; 15 g: *F*_time(5,50)_ = 4.478, *P* = 0.0019; *F*_treatment(1,10)_ = 6.605, *P* = 0.0279; *F*_time×treatment(5,50)_ = 3.365, *P* = 0.0107). In sharp contrast, systemic treatment of sertraline (10 mg/kg), a selective 5-HT reuptake inhibitor, failed to change the thermal and the mechanical thresholds in 6-OHDA-lesioned rats (Figures 7(e) and 7(f)). Thus, it was suggested that the analgesic effect of duloxetine was mainly dependent on the increased NE level in 6-OHDA-lesioned rats.

### 3.8. Inhibitory Effects of Systemic Administration of L-DOPA and Pramipexole on Pain Hypersensitivity in 6-OHDA-Lesioned Rats

**Figure 8:**
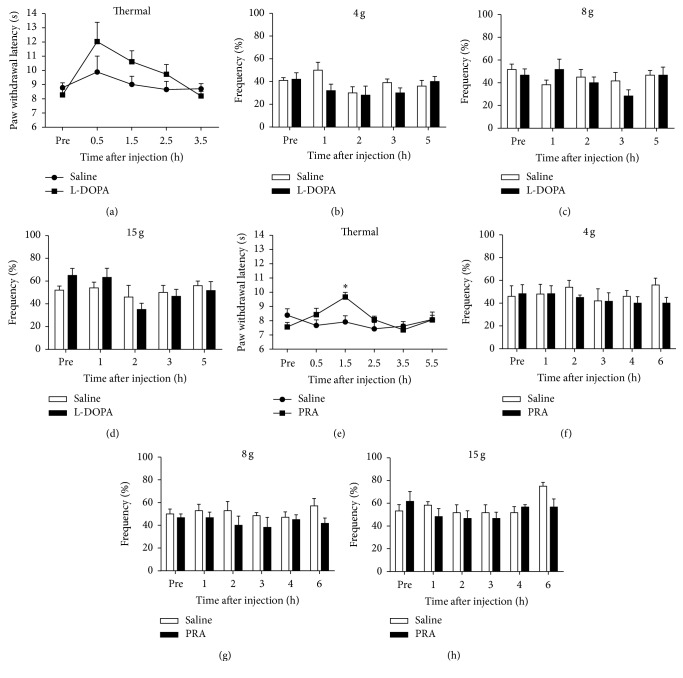
Effects of L-DOPA and pramipexole on thermal and mechanical hypersensitivity in 6-OHDA-lesioned rats. On the 5th week after microinjection of 6-OHDA, (a) systemic application of L-DOPA (15 mg/kg, i.p.) did not significantly inhibit thermal (a) and mechanical hypersensitivity ((b)–(d)) in the 6-OHDA-lesioned rats, although there was a trend to increase the paw thermal withdrawal threshold (*n* = 5-6 for each group). ((e)–(h)) Systemic application of pramipexole (PRA; 1 mg/kg, i.p.) significantly increased the paw thermal withdrawal threshold (e) after injection 1.5 h, but it had no effects on mechanical hypersensitivity ((f)–(h)) in the 6-OHDA-lesioned rats (^*∗*^ *P* < 0.05 compared to the saline control, *n* = 5–7 for each group).

We finally examined the possible pain modulatory effects of drugs targeting dopaminergic system in 6-OHDA-lesioned rats, including Madopar (L-DOPA) and dopamine D2/D3 receptors agonist pramipexole. L-DOPA as a dopamine precursor is one of the most effective pharmacological therapies for the management of PD. Systemic administration of L-DOPA (15 mg/kg, i.p.) slightly increased the paw withdrawal latency to thermal stimulation in 6-OHDA-lesioned rats at the 5th week after surgery, but did not reach statistical significance (Figure 8(a)). L-DOPA failed to change the mechanical hypersensitivity in 6-OHDA-lesioned rats (Figures 8(b)–8(d)). In addition, systemic treatment of a D2/D3 agonist pramipexole (PRA, 1 mg/kg, i.p.), which is widely used for treatment of nonmotor symptoms in PD [7], significantly increased the paw withdrawal latency to thermal stimulation (Figure 8(e)) (*F*_time(5,65)_ = 2.637, *P* = 0.0312; *F*_treatment(1,13)_ = 1.51, *P* > 0.05; *F*_time×treatment(5,65)_ = 847, *P* = 0.0219). However, pramipexole treatment failed to affect mechanical hypersensitivity in 6-OHDA-lesioned rats at the 5th week after surgery (Figures 8(f)–8(h)).

## 4. Discussion

In the present study, we established a PD rat model by stereotaxic infusion of 6-OHDA into the bilateral striatum (CPu) and validated it by TH-immunostaining in the substantia nigra and behavioral analysis. PD rats showed thermal and mechanical hypersensitivity, which were developed at the 4th week after surgery. HPLC analysis showed that NE content, but not dopamine or 5-HT, significant decreased in lumbar spinal cord from PD rats. Additional noradrenergic depletion by injection of DSP-4 aggravated pain hypersensitivity in PD rats. At the 5th week after injection of 6-OHDA, systemic treatment with NE precursor droxidopa or *α*2AR agonist clonidine significantly attenuated pain hypersensitivity in PD rats. Furthermore, application of NE and 5-HT reuptake inhibitors duloxetine, but not 5-HT selective reuptake inhibitors sertraline, significantly inhibited pain hypersensitivity in PD rats. Systemic administration of L-DOPA or the D2/D3 agonist pramipexole slightly inhibited the thermal but not mechanical hypersensitivity in PD rats. Thus, the present study revealed that impairment of descending noradrenergic system plays a key role in PD-associated pain and provided a novel strategy by restoring spinal noradrenergic inhibitory tone to alleviate PD-associated pain.

The suitable PD animal models are essential to elucidate the molecular and cellular mechanisms underlying PD-associated pain [53]. The animal models used for studying PD-associated pain are still limited, such as 1-methyl-4-phenyl-1,2,3,6-tetrahydropyridine- (MPTP-) induced PD mouse model [54] and unilateral [55] or bilateral [48, 49] administration of 6-OHDA-lesioned nigrostriatal pathway in rats. 6-OHDA is highly specific neurotoxin targeted catecholamine neurons though the dopamine active transporter (DAT) [21]. Herein, we used the bilateral stereotaxic injection of 6-OHDA into CPu to produce lesions of nigrostriatal pathway [56] to induce dopaminergic denervation and subsequent cell loss in SNc [57]. In contrast, injection of 6-OHDA into the medial forebrain bundle (MFB) and SNC could result in a rapid loss of the nigrostriatal dopamine (DA) neurons accompanied by severe motor deficits [57], which may interrupt the measurement of pain behaviors. It is considered that bilateral administration of 6-OHDA is closer to human pathology and should be preferred to the unilateral one [49]. In the present study, our immunostaining results showed that TH-positive cells were significantly decreased in the SN and western blotting results confirmed that the protein level of TH expression in the CPu was also significantly reduced. In addition, the motor function of 6-OHDA-lesioned rats evaluated by rotarod test was significantly impaired at the 2nd week after surgery. We next assessed the changes of thermal and mechanical pain threshold by using Hargreaves test [44] and von Frey filament test [45]. Our study have shown that pain hypersensitivity to thermal and mechanical stimulation appeared up to the 4th and 5th weeks after surgery, while the motor symptoms occurred as early as 2nd week after surgery. Pain is the most bothersome nonmotor symptom in early-stage PD patients and even before the appearance of motor symptoms [12]. Notably, we observed that pain hypersensitivity appeared following motor dysfunction in 6-OHDA-lesioned rats, possibly due to the differences between the 6-OHDA-induced PD animal model and PD patients.

We subsequently investigated the mechanisms underlying PD-associated pain in 6-OHDA-lesioned rats. Interestingly, HPLC analysis showed that NE level, but not dopamine or 5-HT, was decreased in spinal cord of 6-OHDA-lesioned rats at the 5th week after surgery. Consistently, early clinical observation also showed degeneration of noradrenergic and serotonergic, but not dopaminergic neurons, in the lumbar spinal cord of parkinsonian patients [58]. The presence of Lewy pathology and loss of cells in the locus coeruleus (LC) was noted to appear almost always in PD patients [28], and loss of NE was even earlier and of greater extent than loss of DA in PD patients [29]. Deficiency of NE already accounts for many features of autonomic impairments, such as orthostatic and postprandial hypotension and some other nonmotor symptoms, such as cognitive impairment, sleep impairment, and depression [26, 30, 31]. However, the role of NE deficiency in PD-associated pain is not investigated. It is well acknowledged that the descending noradrenergic system serves as a principal endogenous analgesic mechanism in the descending pain modulatory system [32, 34]. Thus, we hypothesized that NE deficiency may contribute to PD-associated pain in rats.

In a previous study, the impairment of NE system by administration of DSP-4, which is a competitive inhibitor of NE uptake, selectively caused degeneration of noradrenergic neurons in the locus coeruleus (LC) and potently decreased the nociceptive threshold by using a hot-plate and tail-flick tests [40]. We also found that intracerebroventricular injection of DSP-4 for depletion of NE in the brain was able to decrease the paw withdrawal latency of thermal stimulation and elevated the mechanical sensitivity. Some clinical studies have shown that PD patients with loss of NE in the LC tend to exhibit more pronounced PD nonmotor symptoms (depression, sleep disturbances, etc.) [26, 27], and these symptoms could be alleviated by the reboxetine, a selective NE reuptake inhibitor [59]. However, there have been few studies focused on the role of NE deficiency in PD-associated pain. In our study, when we additionally lesioned NE system by DSP-4, the pain hypersensitivity of PD rats became more earlier and apparent, suggesting that additional NE loss exacerbates pain hypersensitivity in 6-OHDA-lesioned rats. In sharp contrast, when we additionally impaired serotonergic system by intracerebroventricular injection of 5,7-DHT, the pain hypersensitivity of PD rats were not significantly affected. The descending serotonergic system has been shown to either facilitate or inhibit pain processing in spinal cord [60]. The role of descending serotonergic system in PD-associated pain hypersensitivity may be complex and warrants further investigation.

We further asked whether restoring descending noradrenergic inhibitory system could alleviate PD-associated pain. Firstly, we found that thermal and mechanical hypersensitivity could be inhibited by systemic injection of droxidopa in the 6-OHDA-lesioned rats. Droxidopa, namely, L-threo-3,4-dihydroxyphenylserine (L-DOPS), is a pharmacologically inactive NE precursor and can be converted to NE and enter the brain through the blood-brain barrier to increase brain levels of NE. Droxidopa is already used to improve neurogenic orthostatic hypotension symptoms [52]. Thus, the analgesic effects of droxidopa in 6-OHDA-lesioned rats may be attributed to the enhanced level of NE in central nervous system. We next tested the effects of clonidine, which is an *α*2-adrenoreceptor (*α*2AR) agonist, on pain hypersensitivity in 6-OHDA-lesioned rats. Systemic application of clonidine could relieve pain in 6-OHDA-lesioned rats. The previous study has shown the neuroprotection by NE could be through *α*2AR [43]. Previous study also demonstrated that administration of *α*2AR antagonist suppressed the antiallodynic effects of reboxetine, a selective NE reuptake inhibitor, in neuropathic pain rats [36]. Thus, elevated NE level may produce antinociceptive effect possibly via the activation of *α*2AR in the spinal cord of PD rats. However, the essential role of other NE receptor subtypes, such as *α*1AR and *β*AR, in spinal noradrenergic inhibitory tone needs further investigation.

We next tested another way to elevate NE level in the brain for treatment of PD-associated pain by using duloxetine, which is a NE and 5-HT reuptake inhibitor. Duloxetine was shown to be effective for the treatment of neuropathic pain [61, 62]. In our study, systemic injection of duloxetine significantly inhibited pain hypersensitivity in 6-OHDA-lesioned rats at the 5th week after surgery. In sharp contrast, systemic injection of sertraline, which is a selective 5-HT reuptake inhibitor, had no significant effects on pain-related behavior in 6-OHDA-lesioned rats. Thus, the analgesic effect of duloxetine in the 6-OHDA-treated rats may mainly dependent on the NE system, but not 5-HT system.

Finally, we investigated the effects of levodopa (L-DOPA) and dopamine D2/D3 agonist pramipexole on PD-associated pain in rats. L-DOPA was most widely used for the treatment of motor symptoms of PD; however, it has limited effect on nonmotor symptoms [6]. In a previous report, L-DOPA significantly raised pain threshold in PD patients [63]. Interestingly, we found that systemic injection of L-DOPA had a trend to increase the thermal threshold in 6-OHDA-lesioned rats but did not reach significance. Notably, although endogenous dopamine is the direct precursor of NE [64], L-DOPA can increase dopamine levels in the peripheral circulation and the central nervous system but does not increase the concentration of NE [65]. Systemic application of dopamine D2/D3 agonist pramipexole could significantly inhibit the thermal pain hypersensitivity, but not mechanical hypersensitivity. Thus, our results suggested targeting dopaminergic system by L-DOPA and pramipexole may reduce thermal, but not mechanical, pain hypersensitivity in PD rats.

In summary, we found that 6-OHDA-lesioned rats developed pain hypersensitivity and decreased NE level in spinal cord at the 5th week after surgery. Restoring the descending noradrenergic inhibitory tone by droxidopa and duloxetine could alleviate pain hypersensitivity in the 6-OHDA-lesioned rats. These findings suggested that NE deficiency play a key role in the PD-associated pain and restoring the descending noradrenergic inhibitory tone could be considered as an analgesic therapy in PD patients.
